# Molecular and functional characterization of urine‐derived podocytes from patients with Alport syndrome

**DOI:** 10.1002/path.5496

**Published:** 2020-08-19

**Authors:** Corinne Iampietro, Linda Bellucci, Fanny O Arcolino, Maddalena Arigoni, Luca Alessandri, Yonathan Gomez, Elli Papadimitriou, Raffaele A Calogero, Enrico Cocchi, Lambertus Van Den Heuvel, Elena Levtchenko, Benedetta Bussolati

**Affiliations:** ^1^ Department of Molecular Biotechnology and Health Sciences University of Torino Torino Italy; ^2^ Laboratory of Pediatric Nephrology, Department of Development & Regeneration University of Leuven Leuven Belgium; ^3^ Department of Pediatric Nephrology University of Torino Torino Italy; ^4^ Division of Nephrology and Center for Precision Medicine and Genomics, Department of Medicine Columbia University New York NY USA; ^5^ Department of Pediatric Nephrology University Hospitals Leuven Leuven Belgium

**Keywords:** Alport syndrome, genetic defects, collagen IV, urine‐derived podocytes, glomerular endothelial cells, co‐culture, permeability

## Abstract

Alport syndrome (AS) is a genetic disorder involving mutations in the genes encoding collagen IV α3, α4 or α5 chains, resulting in the impairment of glomerular basement membrane. Podocytes are responsible for production and correct assembly of collagen IV isoforms; however, data on the phenotypic characteristics of human AS podocytes and their functional alterations are currently limited. The evident loss of viable podocytes into the urine of patients with active glomerular disease enables their isolation in a non‐invasive way. Here we isolated, immortalized, and subcloned podocytes from the urine of three different AS patients for molecular and functional characterization. AS podocytes expressed a typical podocyte signature and showed a collagen IV profile reflecting each patient's mutation. Furthermore, RNA‐sequencing analysis revealed 348 genes differentially expressed in AS podocytes compared with control podocytes. Gene Ontology analysis underlined the enrichment in genes involved in cell motility, adhesion, survival, and angiogenesis. In parallel, AS podocytes displayed reduced motility. Finally, a functional permeability assay, using a podocyte–glomerular endothelial cell co‐culture system, was established and AS podocyte co‐cultures showed a significantly higher permeability of albumin compared to control podocyte co‐cultures, in both static and dynamic conditions under continuous perfusion. In conclusion, our data provide a molecular characterization of immortalized AS podocytes, highlighting alterations in several biological processes related to extracellular matrix remodelling. Moreover, we have established an *in vitro* model to reproduce the altered podocyte permeability observed in patients with AS. © 2020 The Authors. The *Journal of Pathology* published by John Wiley & Sons Ltd on behalf of Pathological Society of Great Britain and Ireland..

## Introduction

Alport syndrome (AS) is a genetic disorder that affects one in every 50 000 newborns. Around 80% of AS families have X‐linked inheritance caused by mutations in the *COL4A5* gene, while the others have autosomal recessive, or less commonly autosomal dominant, disease, caused by mutations in *COL4A3* or *COL4A4* genes [1]. These genes encode for three of the six different collagen IV α‐chains (α1–α6), which are assembled in type IV collagen networks [2, 3]. Collagen IV α3α4α5 heterotrimer is the main component of the basal membrane in glomeruli, as well as in the cochlea, lens capsule, and retina [4, 5]. Mutations in any one of the three genes lead to the absence of the collagen IV α3α4α5 network, resulting in typical AS clinical features: kidney disease, hearing loss, and eye abnormalities [1].

In particular, the absence of a correct collagen IV α3α4α5 network impairs the functional properties of the glomerular filtration barrier [6]. During nephron maturation, podocytes express, assemble, and secrete collagen IV α3α4α5 proteins that replace the initial embryonic collagen IV α1α1α2 network in the glomerular basal membrane (GBM) [7]. After this developmental switch, the resulting mature GBM is a highly organized 300–400 nm thick collagen IV network [8]. In AS patients, alterations of the GBM, presenting as lamellation of the lamina densa, affect the function of podocytes, which appear to be the first altered cell type. AS podocytes demonstrate foot process effacement, resulting in impaired filtration of blood and passage of blood and/or proteins into the urine [9, 10]. Likewise, in murine models of AS disease (*Col4a3* ^+/−^ and *Col4a3* ^−/−^ mice), podocytes appear with specific morphological alterations characterized by long filamentous and round shaped projections as compared to the podocytes of wild‐type mice [11].

Since in glomeruli, the production of the collagen IV α3α4α5 heterotrimer is restricted to podocytes [12], their involvement in AS pathogenesis is of great interest. Recently, the analysis of kidney biopsies of AS patients clearly showed that podocyte loss starts as early as birth and results in the progressive reduction of podocyte number per glomerulus with time, correlating with overall renal damage [13]. In particular, podocyte number per glomerulus and podocyte nuclear density are significantly reduced in AS patients [14]. However, data on the phenotypic characteristics of human AS podocytes and their functional alterations are currently limited.

Several studies demonstrated that podocyte excretion is a natural phenomenon which precedes proteinuria [15, 16] and that it might be a sensitive marker of glomerular damage [16, 17, 18, 19]. Importantly, this phenomenon could also offer the possibility to isolate podocytes from normal subjects and patients with glomerular disorders [16]. Recently, podocytes isolated from the urine of AS patients have been used for genetic characterization of mutations [20]. However, the restricted cell doubling of primary AS podocytes limits their use as an *in vitro* model necessary for the characterization of their morphological, molecular, and functional profile.

The goal of our study was to set up a novel human *in vitro* model to evaluate possible functional and molecular alterations in human AS podocytes. Urine‐derived podocytes from AS patients were isolated, immortalized, and cloned, and their gene expression profile was characterized by RNA sequencing, in comparison with normal urine‐derived podocytes. Moreover, a three‐dimensional system of podocyte–glomerular endothelial cell (GEC) co‐culture was established to analyse the selective filtration of AS podocytes, compared with control podocytes. Permeability was measured in both static and dynamic co‐culture conditions, confirming the defective filtration observed in patients with AS.

## Materials and methods

### Patients

A total of three patients diagnosed with AS were recruited in this study. All parents of the AS patients provided informed signed consent for urine collection and subsequent experiments, and the Ethics Committee of the University Hospital Leuven approved the research protocol (S54695). The diagnosis was based on measuring creatinine levels and estimated glomerular filtration rate (eGFR) and was confirmed by the characterization of the mutated Alport syndrome genes (Table 1). Patient 1 had decreased eGFR [chronic kidney disease (CKD) stage 2], while patients 2 and 3 showed normal kidney function. All patients had proteinuria and were treated with enalapril. Patients 1 and 3 had perceptive hearing loss; patient 2 had normal hearing. Urine‐derived podocytes were also isolated from a young healthy donor (Table 1).

**Table 1:** Clinical features of control and AS patients.

Patient	Age (years)	Sex	Mutated gene	Mutation type	Mutation	Protein/creatinine (g/g)	eGFR (ml/min per 1.73 m^2^)	Treatment
AS 1	14	F	*COL4A3*	Compound heterozygote	Maternal: p.(Glu647Argfs*45) c.1937dup Paternal: p.(Gly1602Alafs*13) c.4803del	0.47	71	Enalapril
AS 2	11	F	*COL4A5*	Heterozygote	p.(Gly926Alafs*70) c.2777del	0.6	135	Enalapril
AS 3	22	M	*COL4A5*	Hemizygote	p.Gly849Glu (c.2546G>A)	0.36	122	Enalapril
Control	11	F	–	–	–	Neg	–	–

eGFR: estimated glomerular filtration rate.

Normal protein/creatinine ratio: 0.2 g/g; normal eGFR: ≥ 90 ml/min per 1.73 m^2^; control: healthy urine donor.

### Generation of podocyte cell lines

Freshly collected urine was centrifuged at 200 × *g* for 10 min and the pellet was resuspended in DMEM/F‐12 (Life Technologies, Carlsbad, CA, USA) supplemented with 10% FCS (Invitrogen, Carlsbad, CA, USA), 50 IU/ml penicillin, 50 g/ml streptomycin, 5 mm glutamine, 5 g/ml insulin, 5 g/ml transferrin, and 5 mg/ml selenium (all from Sigma‐Aldrich, St Louis, MO, USA). Cells (AS and control podocytes) were immortalized and subcloned using a temperature‐sensitive Simian virus 40 large T (SV40T) and human telomerase reverse transcriptase vectors, as described previously [21]. AB kidney‐derived podocytes, immortalized using the same protocol and kindly gifted by MA Saleem, were used as an additional control [21].

Control and AS podocytes were grown at 33 °C and transferred to 37 °C for 10–14 days to obtain fully differentiated podocytes. Details are provided in supplementary material, Supplementary materials and methods.

### Purification of glomerular endothelial cells (GECs)

Primary normal glomerular microvascular endothelial cells were previously obtained from cell outgrowths of human glomeruli and CD31 sorting, and characterized by morphology and expression of a panel of endothelial antigens [22] (see supplementary material, Supplementary materials and methods and Figure S1).

### RNA extraction and RT‐qPCR

RNA was extracted using Trizol (Life Technologies) according to the manufacturer's protocol. Aliquots of total RNA (200 ng) were reverse‐transcribed using a High Capacity cDNA Reverse Transcription Kit (Applied Biosystems, Foster City, CA, USA). Levels of mRNA were assessed by qPCR, using a mix containing 5 ng of cDNA, 100 nm of each primer (see supplementary material, Supplementary materials and methods), and 1× SYBR Green PCR Master Mix (Applied Biosystems), and assembled into a 96‐well StepOne Real Time System (Applied Biosystems). Negative cDNA controls were cycled in parallel in each run. Data are shown as relative quantification (2^−ΔΔCt^). *GAPDH* expression was used to normalize cDNA inputs. Sample similarity plots were generated using Morpheus (https://software.broadinstitute.org/morpheus/).

### Protein extraction and western blotting

Cell pellets were lysed at 4 °C for 15 min in RIPA buffer (Sigma) supplemented with protease and phosphatase inhibitors. Details are provided in supplementary material, Supplementary materials and methods.

### ELISA assays

The concentrations of human VEGFA, Col4α3, and Col4α5 in protein lysate and supernatant of differentiated podocytes were measured using ELISA kits according to the manufacturer's instructions (*n* = 3 experiments in duplicate). Details may be found in supplementary material, Supplementary materials and methods.

### RNA sequencing and analysis

Details are presented in supplementary material, Supplementary materials and methods. RNA‐seq data have been deposited in the GEO database (http://www.ncbi.nlm.nih.gov/projects/geo/) under the GEO accession number GSE134011.

### Single‐cell tracking assay

For each condition, 2000 differentiated cells per well were seeded in a polystyrene, transparent 96‐well plate, 6 h prior to the assay, in triplicate, as described previously [23]. For 12 h, each well was imaged every 5 min at the same coordinates using 10× magnification. Live imaging was performed on a Zeiss LSM 880 Airyscan microscope (Carl Zeiss Microscopy GmbH, Oberkochen, Germany). Microscopy images were further processed and analysed using Zeiss ZEN imaging software (Carl Zeiss, Jena, Germany) and Fiji/ImageJ free software. In brief, ten cells were tracked per well, yielding 30 tracked cells per cell line. The total distance travelled and the speed of the individual cells were recorded.

### Immunofluorescence

Immunostaining was performed on cells co‐cultured for 48 h on an insert, fixed in 4% paraformaldehyde. Imaging was performed using a Leica TCS SP5 confocal system (Leica Microsystems S.r.l., Wetzlar, Germany). Details are provided in supplementary material, Supplementary materials and methods.

### Three‐dimensional co‐culture assembly

For all co‐culture experiments, GECs were seeded first on the lower PET membrane side at a density of 8 × 10^4^ cells per 12‐well insert (static co‐culture: 3 μm pore size, Falcon‐Corning, Glendale, AZ, USA; dynamic co‐culture: 0.45 μm pore size, ipCELLCULTURE^TM^ Track Etched Membrane, it4ip S.A., Louvain‐la‐Neuve, Belgium) and, after 6 h, podocytes were seeded on the upper side at the same density. Each cell type was cultured in its growth medium for 48 h before the permeability assay and medium were changed once the day after cell plating.

### Millifluidic system

For dynamic experiments, we used a millifluidic device, fabricated by IVTech Srl (Lucca, Italy), consisting of two independent circuits that work in parallel. During permeability tests, the medium was maintained with a flow rate of 100 μl/min and cells in the chamber were subjected to a shear stress of 8 × 10^−5^ dyn/cm^2^. To roughly calculate this value, the following equation was used: τ = 6μ*Q*/*bh* ^2^, where μ is the medium viscosity (g/cm per s), *Q* is the volumetric flow rate (cm^3^/s), *b* is the channel width, and *h* is the channel height [24, 25, 26].

### Permeability assays

Permeability assays were performed after 48 h of co‐culture by measuring BSA filtration. Complete medium (1 ml) containing or not FITC‐BSA (1 mg/ml, Sigma) was placed in the lower endothelial and upper podocyte compartments, respectively. To measure the podocyte filtration ability in basal to apical direction, 100 μl of medium was taken after 6 h from the podocyte compartment and the passage of FITC‐BSA was determined by fluorimetry in triplicate. For the dynamic system, podocyte medium enriched with FITC‐BSA (1 mg/ml) was circulated into the endothelial compartment, while normal culture medium circulated into the upper compartment. After 3 h of perfusion, the passage of BSA‐FITC across the barrier was determined by sampling of fluid from the upper channel. FITC signal was measured in triplicates using a fluorimeter.

### Statistics

Data are shown as mean ± SD. At least three replicates were performed for each experiment. Two‐tailed Student's *t*‐tests were used for analysis when two groups of data were compared, while two‐way ANOVA with Dunnett's multiple comparison test was applied when comparing more than two groups of data. All statistical analyses were performed using GraphPad Prism software version 6.0 (GraphPad Software, Inc, San Diego, CA, USA). *p* < 0.05 was considered significant.

## Results

### Generation of conditionally immortalized podocyte lines from urine of patients with AS

We generated conditionally immortalized podocytes from the urine of three different patients with AS (aged 16.5 ± 5.5 years) and from a normal subject (aged 11 years). AS patient 1 showed compound heterozygous mutations in the *COL4A3* gene, while AS patients 2 and 3 had mutations in the *COL4A5* gene in heterozygosity and in hemizygosity, respectively (Table 1). All AS patients were characterized by an altered protein/creatinine ratio (0.48 ± 0.12 g/g), but only patient 1 presented a mildly reduced eGFR (Table 1).

**Figure 1:**
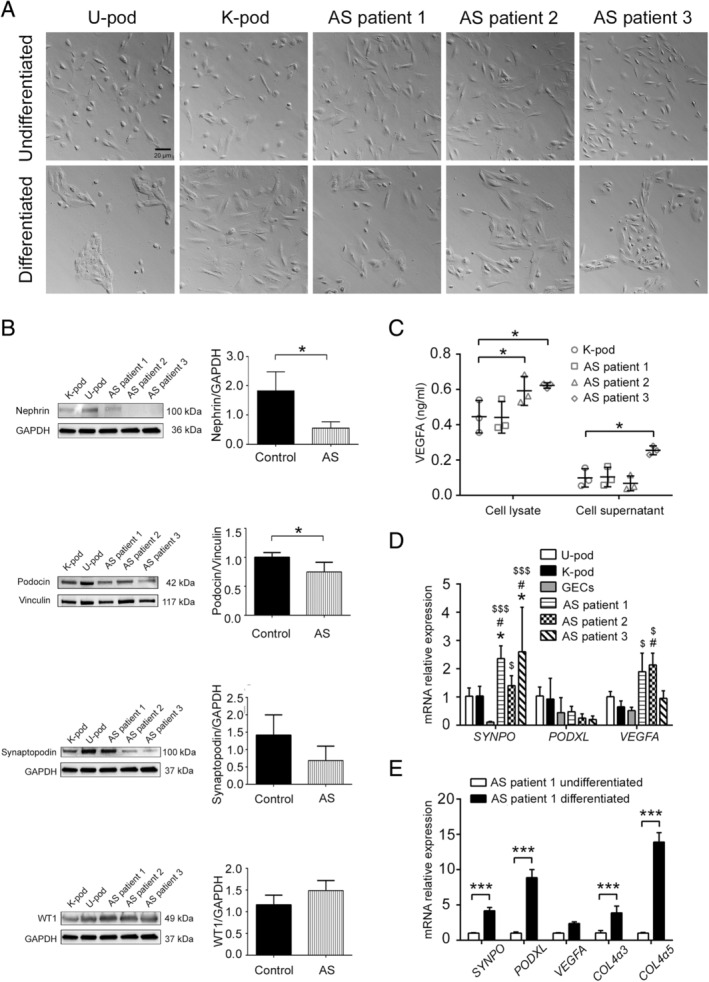
Morphology and marker expression of cultured AS podocytes. (A) Representative light microscopy images of undifferentiated (upper panels) and differentiated (lower panels) podocytes from normal urine (U‐pod), normal kidney (K‐pod), and AS patient urine. Original magnification: ×20; scale bar: 20 μm. (B) Western blot analysis (representative images and quantification) of nephrin, podocin, synaptopodin, and WT1 of differentiated control (U‐pod and K‐pod) and AS podocytes. Data are expressed as mean ± SD of band intensity normalized to vinculin or GAPDH of three different experiments. *p < 0.05. (C) VEGFA ELISA performed on cell lysates and cell supernatants obtained from differentiated AS podocytes, compared with control (K‐pod). Data are mean ± SD of three different experiments. *p < 0.05. (D) RT‐qPCR for synaptopodin (*SYNPO*), podocalyxin (*PODXL*), and *VEGFA* transcripts. Data are shown as relative quantification, normalized to *GAPDH* and to control podocytes (U‐pod). Three clones for each AS patient were used and data are expressed as mean ± SD of three different experiments. GECs were used as a negative control. *p < 0.05 AS patient podocytes versus U‐pod; ^#^ *p* < 0.05 AS patient podocytes versus kidney‐derived podocytes (K‐pod); ^$^ *p* < 0.05 and ^$$$^ *p* < 0.0001 AS patient podocytes versus GECs (negative control). (E) RT‐qPCR for podocyte marker transcripts (*SYNPO, PODXL, VEGFA*) and collagen IV isoforms (*COL4A3*, *COL4A5*) of AS patient 1 podocytes before and after differentiation. Data shown as relative quantification, normalized to *GAPDH* and to undifferentiated AS patient 1 podocytes, expressed as mean ± SD. ***p < 0.0001.

Primary urine‐derived podocytes were immortalized by inducing the expression of temperature‐sensitive SV40T and telomerase reverse transcriptase allowing growth at 33 °C and differentiation at 37 °C [21]. Nine clones were picked for each patient and were subsequently grown as separate cell lines. Conditionally immortalized podocytes previously obtained from a normal renal biopsy and characterized [21] were used as an additional control. At the permissive temperature of 33 °C, AS podocytes proliferate in a comparable way to normal urine‐derived and kidney‐derived podocytes, used as controls (Figure 1A). After 10–14 days at 37 °C, differentiated cells showed an irregular and enlarged cell body with formation of protrusions very similar to controls (Figure 1A).

### Podocyte marker expression in differentiated AS podocytes

To confirm the podocyte feature of AS podocytes, three clones for each AS patient were analysed for the expression of the specific podocyte markers synaptopodin, podocalyxin, nephrin, podocin, WT1, and VEGFA. All markers were expressed by AS podocytes, although a reduction in some adhesion molecules in relation to urinary and tissue control podocytes was observed, in line with previous reports [1, 27]. In particular, levels of nephrin and podocin were reduced in AS podocytes compared with controls (Figure 1B), while synaptopodin showed only a trend for reduction. No differences were observed in WT1 expression. Increased expression of VEGFA was also found in AS patients 2 and 3 (Figure 1C). *VEGFA* transcript levels were also significantly increased in AS patient 2 compared with controls. AS podocytes also expressed lower levels of *SYNPO* mRNA, but not of *PODXL* (Figure 1D). Glomerular endothelial cells were used as a negative control (Figure 1D). The expression of podocyte markers in AS podocytes was significantly increased after 10–14 days of differentiation at 37 °C, in relation to proliferating cells at 33 °C (Figure 1E), as reported for control kidney‐derived podocytes [21].

Taken together, these results indicate that AS podocytes express most of the typical podocyte markers, with a specific reduction of glomerular slit diaphragm proteins.

### Collagen IV isoform expression in differentiated AS podocytes

**Figure 2:**
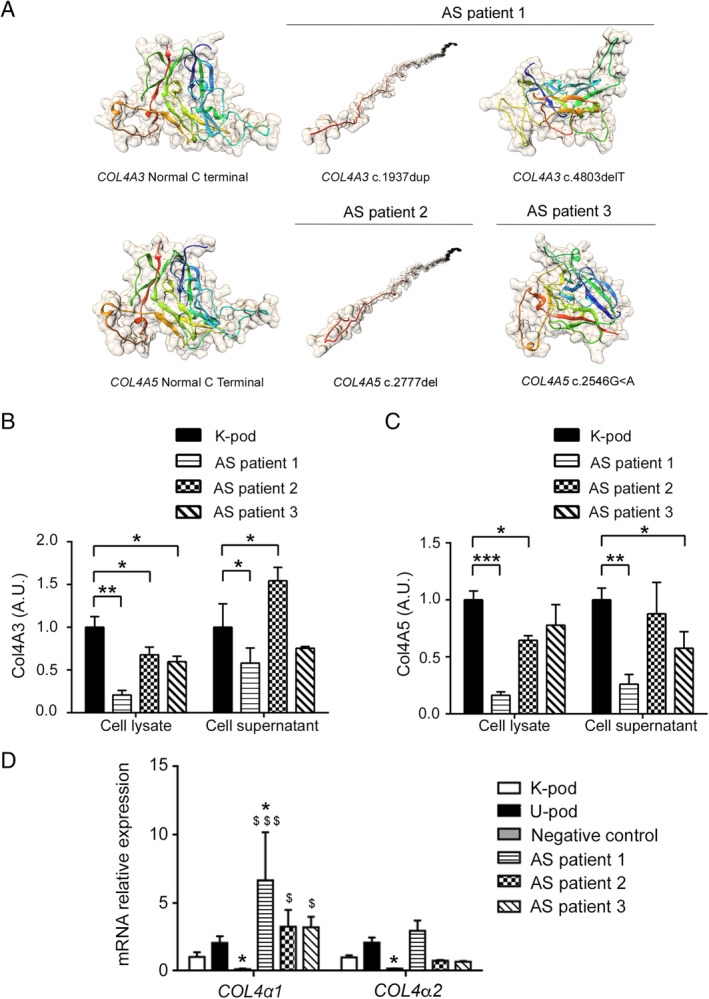
Collagen IV isoform expression in AS podocytes. (A) 3D models of the C‐terminal C4 domains for Col4α3 and Col4α5 in AS patients were generated using *Phyre2* software, as described in supplementary material, Supplementary materials and methods. Images show the structural modifications of COL4α3 and COL4α5 chains predicted on the basis of the different mutations, compared with normal. In patients AS1 and AS2, frameshift mutations cause the protein to be truncated and with predicted alteration in folding (c.1937dup and c.2777del), or conformation (c.4803del). In patient AS3, a single nucleotide variant (c.2546G>A) leads to minor differences in protein folding. (B) COL4α3 ELISA performed on cell lysates and cell supernatants of differentiated AS podocytes and control kidney‐derived podocytes (K‐pod). (C) COL4α5 ELISA performed on cell lysates and cell supernatants of differentiated AS podocytes compared with control kidney‐derived podocytes (K‐pod). ELISA data are expressed as arbitrary units (A.U.) and are the mean ± SD of three different experiments normalized to control (referred to as 1). *p < 0.05; **p < 0.001; ***p < 0.0001. (D) RT‐qPCR for *COL4A1* and *COL4A2* genes. Data are shown as relative quantification, normalized to *GAPDH* and to control kidney‐derived podocytes (K‐pod). Three clones for each AS patient were analysed and data are expressed as mean ± SD. GECs were used as a negative control. ^$^ *p* < 0.05 and ^$$$^ *p* < 0.0001 versus K‐pod; *p < 0.05 versus U‐pod.

In order to better evaluate the collagen IV characteristics in the three different AS patients of our study, we created 3D models of the protein secondary structure of the C‐terminal C4 domains, the terminal portion predictable by 3D modelling. (see supplementary material, Supplementary materials and methods). The models of Col4α3 and Col4α5 represent the normal 3D aspect of the protein C4 terminal domains. AS patient 1 presented frameshift mutations in *COL4A3* leading to a truncated amino‐acid sequence at residues 690 (maternal allele) and 1613 (paternal allele), resulting in alterations of the conformation of C‐terminal C4 residues as shown in Figure 2A. Similarly, frameshift mutation of *COL4A5* in AS patient 2 resulted in a truncated amino‐acid sequence at residue 994, causing the protein to be truncated and unfolded (Figure 2A) from one of the two X chromosomes. In AS patient 3, a single nucleotide variant of *COL4A5* was present, leading to minor differences from the reference sequence, as shown in Figure 2A.

We subsequently analysed the expression of collagen IV isoforms in three clones for each patient. At protein level, *COL4A3* gene‐mutated podocytes from AS patient 1 showed a significant reduction of COL4α3 protein in both the cell lysate and the supernatant compared with control (Figure 2B). In addition, COL4α5 protein was also reduced (Figure 2B). In parallel, podocytes from AS patients 2 and 3, mutated in the *COL4A5* gene, showed a significant reduction of Col4α5 protein compared with control both in cell lysates and in the supernatant (Figure 2C). A reduction in Col4α5 protein was confirmed in AS patient 3 by confocal microscopy (see supplementary material, Figure S2). COL4a3 protein was also reduced in the cell lysate, but not in the supernatant (Figure 2C), of AS patients 2 and 3, as confirmed at mRNA level in AS patient 2 (see supplementary material, Figure S3).

*COL4A1* and *COL4A2* transcript levels appeared significantly increased in AS patient 1 compared with control podocytes (Figure 2D). These analyses confirm the alterations in collagen IV expression by AS podocyte lines, reflecting disease mutations.

### RNA‐sequencing analysis of differentiated AS podocytes

**Figure 3:**
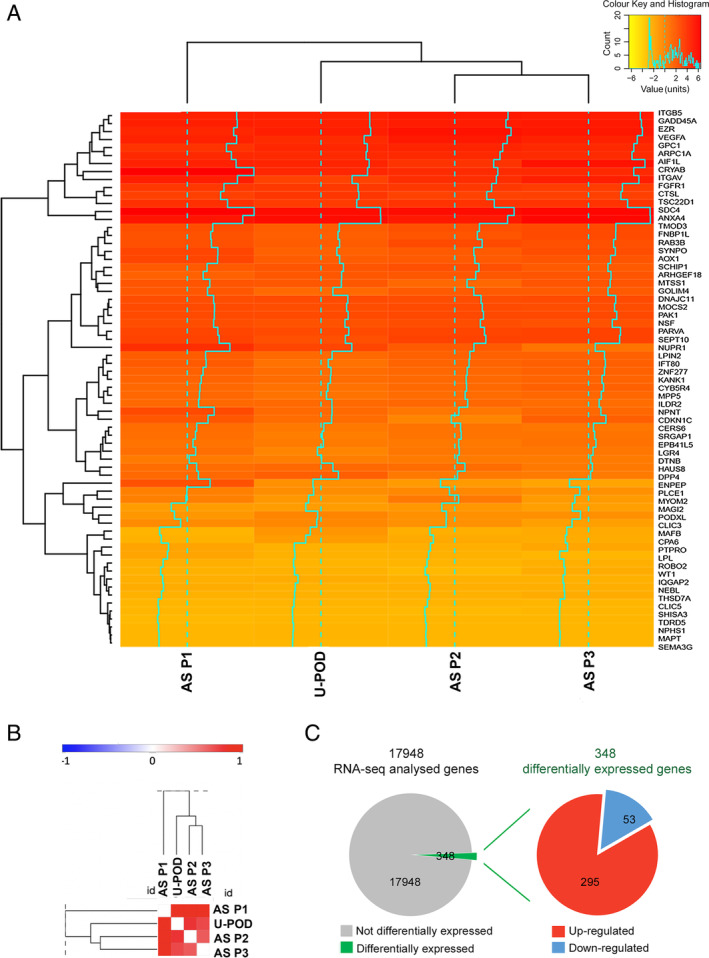
RNA‐seq analysis of AS patients. (A) Heatmap of podocyte signatures showing similar levels of expression of podocyte genes in AS podocytes (three clones per patient) and in control podocytes (U‐pod). (B) Pearson similarity plot of podocyte signature showing similar levels of expression of podocyte genes in AS podocytes (three clones per patient) and in control podocytes (U‐pod). (C) Pie chart representation of up‐regulated (red) and down‐regulated (blue) differentially expressed transcripts in AS patient‐ compared with control urine‐derived podocytes. RNA‐seq was performed on three AS patients (AS P1, P2, P3), including three clones for each patient, and urine‐derived podocytes (U‐pod) as a control.

RNA sequencing was performed on podocytes isolated from urine of the three AS patients to investigate the characteristics of the AS transcriptome, using urine‐derived podocytes as a control. A specific signature composed of 68 podocyte‐typical genes, previously described by Lu *et al* [28], was investigated. AS podocyte lines did not reveal significant differences from the control (Figure 3A,B), confirming the podocyte phenotype of all AS cell lines (three clones per patient).

**Figure 4:**
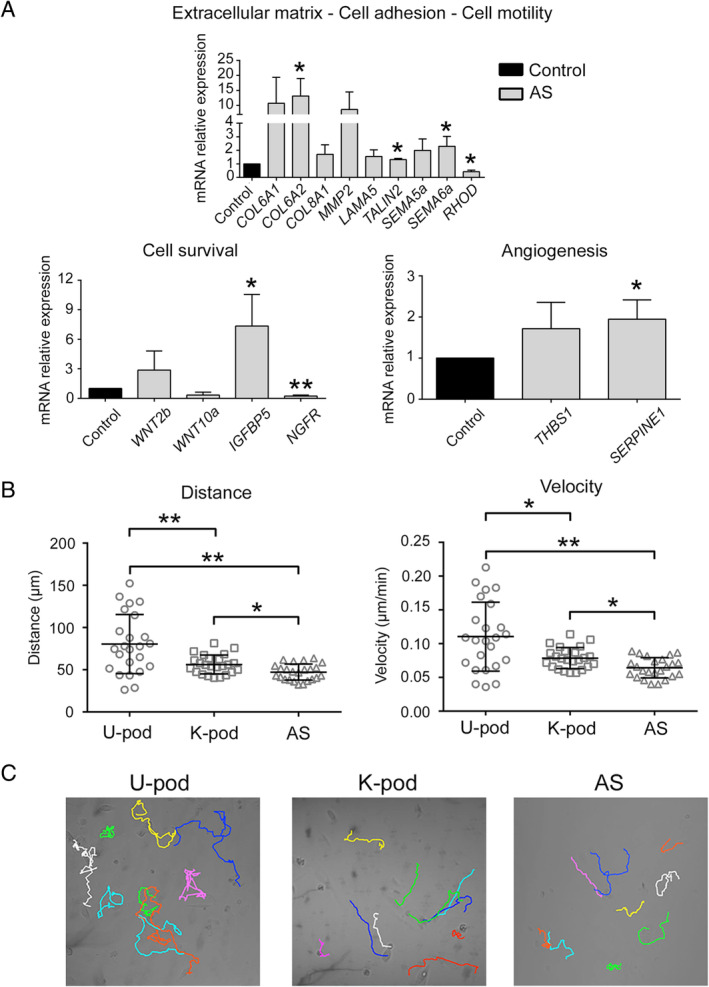
Validation of differentially expressed genes in AS. (A) mRNA expression of genes differentially expressed in AS related to cell motility, survival, and angiogenesis was evaluated by RT‐qPCR. Data are shown as relative quantification, normalized to *GAPDH* and to control kidney‐derived podocytes (K‐pod). One clone for each AS patient was analysed and the results are expressed as mean ± SD of the three AS patients. (B) Single‐cell tracking assay showing reduced motility in AS podocytes compared with urine‐derived podocytes (U‐pod) and kidney‐derived podocytes (K‐pod). Three independent experiments were performed. *p < 0.05; **p < 0.001. (C) Representative images of the cell motility of control podocytes and AS podocytes..

To identify altered gene expression profiles specific for AS podocytes, we analysed the differential expression analysis of the three different AS podocyte lines (three clones per patient) with respect to control podocytes and detected a total of 348 deregulated genes (|log_2_ fold‐change| ≥ 0.8, adjusted *p* ≤ 0.1) out of 17 948 (Figure 3C). Specifically, 295 genes were up‐regulated, while 53 were down‐regulated. To functionally characterize the differentially expressed genes, Gene Ontology (GO) enrichment analysis was performed. GO enrichment analysis of biological processes highlighted the presence of genes involved in morphogenesis and development of a branching epithelium, extracellular matrix and structure organization, cell adhesion, and cellular response to endogenous stimulus (Table 2). Moreover, the GO cellular components confirmed the role of extracellular matrix and adhesion components (Table 2). Using Ingenuity Pathways Analysis (IPA), we observed a high level of connectivity among a large subset of differentially expressed genes in AS podocytes (supplementary material, Figure S4). The differential expression, identified by RNA sequencing, of collagen molecules (*COL6A1*, *COL6A2*, *COL8A1*), *MMP2*, extracellular matrix–actin interactors (*LAMA5*, *TALIN2*, *RHOD*), and adhesion molecules (*SEMA5A*, *SEMA6A*) was confirmed by RT‐qPCR analysis (Figure 4A). Moreover, players of the WNT signalling pathway (*WNT2B*, *WNT10A*) and molecules involved in cell survival (*IGFBP5*, *NGFR*) and angiogenesis (*THBS1, SERPINE1*) were also confirmed as differentially expressed in AS podocytes by RT‐qPCR (Figure 4A).

**Table 2:** Gene Ontology (GO) analysis of differentially expressed genes.

**GO biological process**		ID	Name	*P* value
	1	GO:0060429	Epithelium development	3.618E‐9
	2	GO:0035295	Tube development	1.375E‐8
	3	GO:0061138	Morphogenesis of a branching epithelium	2.574E‐8
	4	GO:0002009	Morphogenesis of an epithelium	2.688E‐8
	5	GO:0007155	Cell adhesion	2.980E‐8
	6	GO:2000026	Regulation of multicellular organismal development	3.537E‐8
	7	GO:0030198	Extracellular matrix organization	3.907E‐8
	8	GO:0022610	Biological adhesion	3.931E‐8
	9	GO:0071495	Cellular response to endogenous stimulus	4.120E‐8
	10	GO:0043062	Extracellular structure organization	4.122E‐8

**GO cellular component**		ID	Name	*P* value
	1	GO:0031012	Extracellular matrix organization	1.062E‐8
	2	GO:0070161	Anchoring junction	1.614E‐5
	3	GO:0045178	Basal part of cell	1.905E‐5
	4	GO:0005912	Adherens junction	2.586E‐5
	5	GO:0030054	Cell junction	1.335E‐4
	6	GO:0005604	Basement membrane	1.600E‐4
	7	GO:0044420	Extracellular matrix component	2.458E‐4
	8	GO:0005615	Extracellular space	1.487E‐4
	9	GO:0005925	Focal adhesion	3.051E‐4
	10	GO:0005815	Microtubule organization centre	3.052E‐4

This table (in two parts) shows the Gene Ontology (GO) analysis of differentially expressed genes in AS patients compared with control urine‐derived podocytes: biological process and cellular component. In each table, the identification (ID) number, the name, and the *P* value associated with the GO are given.

Taken together, these data highlight differential expression in AS podocytes of genes involved in cell adhesion, motility, and survival, with alteration in extracellular matrix and cell junction component biosynthesis, including collagen molecules. We therefore analysed the possible impact of the altered podocyte phenotype on their ability to migrate. As shown in Figure 4B,C, AS podocytes showed reduced motility in terms of speed and distance with respect to controls.

### Static permeability analysis of GEC–podocyte co‐cultures

**Figure 5:**
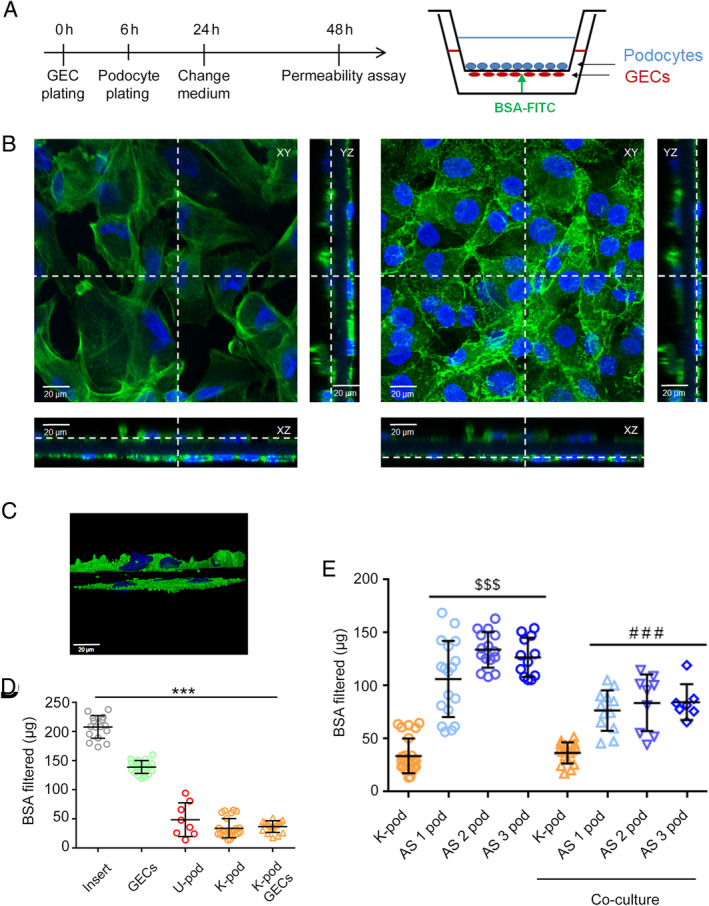
Static permeability assay in podocyte–GEC co‐cultures. (A) Schematic representation of co‐culture and experimental set‐up: podocytes and GECs were co‐cultured for 48 h before the permeability assay was performed, as described in the Materials and methods section. (B) Immunofluorescence of co‐cultures showing the entire field and cross‐sections (*XZ* and *YZ*) of both the upper podocyte layer (left) and the lower GEC layer (right). Cells were stained with phalloidin (green) and nuclear staining was performed with Hoechst dye 33342. Original magnification: ×400. (C) Snapshot of a perspective view of 3D reconstruction of the co‐culture system (see supplementary material, Video S1). (D, E) FITC‐BSA permeability indicating albumin passage from the GEC compartment to the podocyte compartment was measured over 6 h. AS podocytes alone or in co‐culture showed significantly increased filtration compared with control podocytes alone or in co‐culture. Data are expressed as the mean amount of filtered BSA‐FITC of four different experiments using at least three inserts for each condition in each experiment. ***p < 0.0001 insert versus all conditions; ^$$$^ *p* < 0.0001 AS podocytes versus K‐pod; ^###^ *p* < 0.0001 AS podocyte co‐culture versus K‐pod co‐culture. U‐pod: urine‐derived podocytes; K‐pod: kidney‐derived podocytes; AS 1‐2‐3 pod: AS 1‐2‐3 patient podocytes.

To study the functionality of AS podocytes *in vitro*, a co‐culture system that mimics the interaction between GECs and podocytes was assembled. GECs were seeded on the bottom side of PET inserts, and differentiated kidney‐derived or AS podocytes were seeded on the upper side (Figure 5A). The co‐culture obtained consisted of two homogeneous confluent layers of cells separated by a porous PET membrane, as shown by immunofluorescence (Figure 5B,C). The orthogonal view of co‐culture slides shows a single thick layer of differentiated podocytes with typical protrusions, separated by the thin membrane from a single flat layer of GECs (Figure 5B). The 3D reconstruction allowed the inspection, in all three dimensions, of the co‐culture assembly (Figure 5C and supplementary material, Video S1).

A filtration assay was performed by measuring the transit of FITC‐BSA from the lower GEC compartment to the upper podocyte compartment in different experimental conditions (Figure 5D,E). Albumin permeability was significantly reduced when GECs or/and control podocytes were plated on the insert after 6 h of incubation (*p* < 0.0001). In particular, podocytes alone showed a higher resistance to albumin transit through the membrane than GECs alone. Interestingly, the presence of AS podocytes alone significantly increased albumin permeability after 6 h, compared with control podocytes alone (*p* < 0.0001). Finally, the same significantly increased permeability was obtained when GEC–AS podocyte co‐cultures were compared with GEC–control podocyte co‐cultures (*p* < 0.0001), reproducing the defective permeability typical in AS patients. Cell counts of the plated cells showed no significant difference between control and AS podocytes, excluding that the altered filtration of AS podocytes was due to an increased cell detachment.

### Dynamic permeability analysis of GEC–podocyte co‐cultures

**Figure 6:**
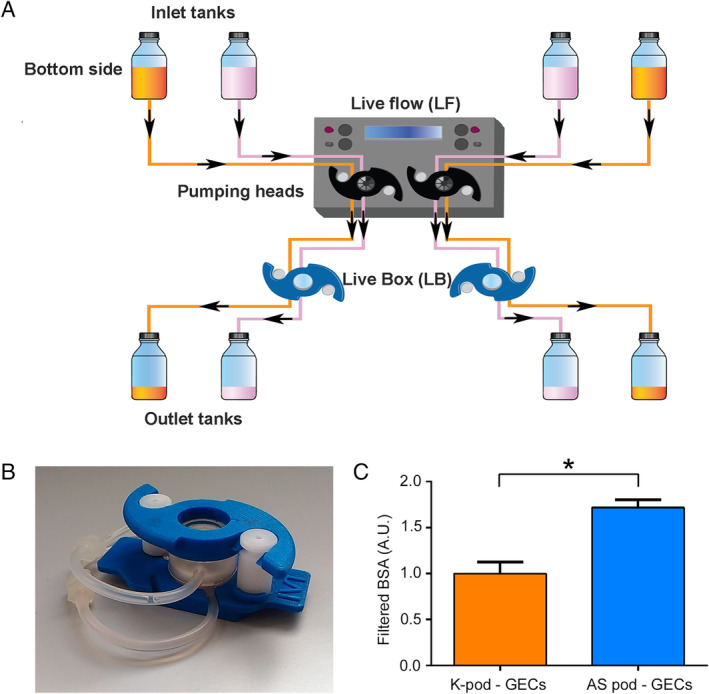
Millifluidic system and permeability assay in podocyte–GEC co‐cultures. (A) Schematic representation of the millifluidic system, as described in the Materials and methods section. Cells seeded in the LiveBox (LB) were perfused with fluids circulated by peristaltic pumps at 100 μl/min in two independent circuits. (B) Representative image of a chamber of LiveBox. (C) After 48 h of podocyte/GEC co‐culture, the passage of FITC‐BSA from the lower to the upper compartment was measured in the dynamic system over 3 h. The permeability in AS co‐cultures was significantly higher than that in control kidney‐derived co‐cultures. Data are expressed as mean ± SD of three different experiments performed in parallel in control and AS podocytes. *p < 0.05. K‐pod: kidney‐derived podocytes.

To analyse the permeability of AS podocytes in dynamic conditions, we adopted a millifluidic system that allows continuous perfusion in co‐culture under 8 × 10^−5^dyn/cm^2^ shear stress (Figure 6A). Cells plated on the PET membrane were maintained in the bioreactor for 48 h before starting the permeability assay (Figure 6B). The albumin filtration rate 3 h after GEC–AS podocyte co‐culture was significantly higher than that in the GEC–control kidney‐derived podocyte co‐culture (Figure 6C, *p* = 0.0012), confirming the increased permeability in AS conditions. The permeability assays were therefore able to recapitulate the AS alterations and confirm from a functional point of view the altered podocyte phenotype demonstrated by molecular analysis.

## Discussion

Increasing evidence indicates that AS podocytes possess an altered phenotype, beyond their altered collagen IV synthesis, which can be involved in the pathogenesis and progression of the disease. In the present study, we isolated and characterized podocytes derived from the urine of three patients with AS, with genetic alterations of either *COL4A3* or *COL4A5*, to assess common pathological features due to collagen IV mutations. AS podocytes showed specific changes in the transcript levels of genes involved in cell motility, adhesion, proliferation, and angiogenesis, with related alterations in their functional properties. In order to study their permeability *in vitro*, we set up a co‐culture system that reproduced the functional aberrations typical of AS.

The availability of human AS podocytes in culture is at present limited. Podocyte‐like cells have been recently obtained from induced pluripotent stem cells (iPS) of AS patients [29, 30] and may represent a promising approach. However, it might be difficult to obtain a pure population for podocyte characterization, as differentiated iPS cultures contained only 30–50% cells with podocyte‐like morphology [29]. Human urine, both from patients and from healthy subjects, has been used previously as a non‐invasive valuable source of podocytes [16]. In patients with active glomerular disease, podocytes are shed from the glomerulus as a response to local environmental factors. Urine‐derived podocytes from both patient and healthy subjects appear positive for podocyte markers, viable, and able to grow in culture [16]. For instance, urine‐derived AS podocytes have been recently obtained and used for genetic studies [20]. In the present study, by exploiting the accelerated podocyte loss in patients with AS [13, 14], we successfully isolated AS podocytes from the urine of three young patients with different collagen IV mutations; conditionally immortalized the primary cells; and generated clonal lines in order to avoid the presence of contaminating non‐podocytic cell types [16]. A similar procedure has been described for urine‐derived podocytes from patients with focal and segmental glomerulosclerosis [27].

The evaluation of a podocyte signature, using a panel of 68 previously described genes [28], demonstrated the podocyte nature of our clonal cell lines. Interestingly, we found down‐regulation of nephrin, podocin, and synaptopodin in AS podocytes, confirming an observation already described in AS kidney biopsies [31]. The relevance of nephrin and synaptopodin levels was previously underlined by their negative correlation with the degree of proteinuria in AS patients [31]. Moreover, we confirmed in each AS podocyte line the alterations in the expression of the mutated collagen IV isoform, confirming that these AS lines are useful for investigating the defective collagen IV protein network and the related podocyte alterations.

Literature data describing the AS podocyte phenotype are quite scarce. Reduced podocyte number per glomerulus and podocyte nuclear density [14] are commonly described in human AS biopsies; however, podocyte hypertrophy is the only cell feature reported [32]. In a murine model of AS, electron microscopy analysis showed the presence of altered podocyte cell bodies with disorganized primary processes and protrusions [11, 33]. From a molecular point of view, the glomerular gene expression profile in a mouse model of AS highlighted induction of MMP‐10 and of the matrix protein mindin, suggesting the involvement of matrix factors in progression [34]. Our study provides evidence of an altered phenotype of AS podocytes, in comparison with control podocytes, common to the three different collagen IV mutations, reflected into altered functions, namely decrease in cell motility and increase in permeability. By gene profiling, we identified restricted and specific alteration of pathways related to basal membrane adhesion, cytoskeleton modulation, and angiogenesis. These data clearly indicate that AS podocytes display altered features related to their interaction with the matrix. We identified 295 up‐regulated and 53 down‐regulated genes as compared to control urine‐derived podocytes. Among differentially expressed genes, we found *MMP2*, whose altered expression was previously described in an AS canine model [35], and *LAMA5*, whose mRNA and protein up‐regulation were shown in AS‐mouse glomeruli [36]. Among the identified genes and pathways differentially expressed in AS podocytes, dysregulated activation of the WNT pathway has been related to podocytopathies and proteinuria [37, 38], while *IGFBP5* has been described as a promoter of epithelial and fibroblast responses in fibrosis [39].

Taken together, the data indicate a general alteration in AS podocytes of matrix interaction and cytoskeletal organization, both necessary to prevent podocyte effacement and maintain permeability control. For instance, reduction of synaptopodin, which in turn controls RhoA activity [40], as well as of cytoskeletal proteins such as vinculin, known to maintain an intact glomerular filtration barrier [41], may induce impairment of cell motility and increase permeability. Further investigation of small GTPase activities in urinary AS podocyte would be important to support the findings.

The observed alterations were reflected by altered podocyte functions, mainly related to cell motility and adhesion. Furthermore, we could reproduce the *in vivo* AS permeability alteration by assembling a co‐culture system of AS podocytes and GECs. In our co‐culture model, we preferred to avoid the addition of an exogenous collagen IV coating, as this GBM component is defective in AS [42]. In this system, we measured the passage of BSA‐FITC from the lower (GEC compartment) to the upper (podocyte compartment) side, which is driven by osmotic forces and is halted by the presence of cellular elements [42, 43]. Similar to the defective glomerular permeability in AS patients [1], AS podocyte–GEC co‐cultures showed significantly increased filtration of albumin compared with the control co‐cultures. To mimic the physiological haemodynamic component, we switched from a static to a dynamic model using a millifluidic device. Also in this dynamic system the permeability in AS podocyte–GEC co‐culture was significantly increased. We are, however, aware that this model does not fully mimic the glomerular alterations observed in AS patients, due to the lack of a glomerular basal membrane. Moreover, the isolation of urinary AS podocytes detached by the glomerular basal membrane in a disease state may introduce a selection bias.

In conclusion, we describe here a new human *in vitro* model that molecularly and functionally reproduces the defective AS podocytes. Using urine‐derived AS podocyte cell lines, we characterized molecular and morphological defects common to collagen IV mutation and related to cell adhesion, motility, survival, and angiogenesis. Moreover, altered permeability measured in AS podocyte–GEC co‐culture underlines the potentiality of this model. In fact, this *in vitro* model could also be used for the screening of pharmacological compounds, which might restore functional properties to AS podocytes.

## Author contributions statement

CI performed characterization and functional experiments, statistical analysis, interpretation of results, and prepared the manuscript and all the figures. EC performed the 3D modelling. LB, FA, YG and EP performed *in vitro* experiments. MA, LA and RC performed RNA sequencing and data analysis. FA, LVDH and EL generated immortalized podocytes and contributed to the study design. BB contributed to the study design, study coordination, and interpretation of the data. All the authors were involved in writing the paper and had final approval of the submitted and published versions.

## Supporting information


**Supplementary materials and methods**

**Figure S1.** Glomerular endothelial cell (GEC) characterization
**Figure S2.** Immunofluorescence microscopy for COL4α5 protein
**Figure S3.** RT‐PCR for *COL4A3* and *COL4A5* transcripts
**Figure S4.** Connectome of differentially expressed genes in AS podocytes

**Video S1.** 3D reconstruction of a podocyte–GEC co‐culture

## Data Availability

RNA‐seq data have been deposited in the GEO database under the GEO accession number GSE134011 (https://www.ncbi.nlm.nih.gov/geo/query/acc.cgi?acc=GSE134011).
