# Supplementary material for: Molecular and functional characterization of urine‐derived podocytes from patients with Alport syndrome
Source: J Pathol. 2020 Aug 19;252(1):e5496. doi: 10.1002/path.5496 (PMC7589231; doi:10.1002/path.5496)
Supplement: Supplementary file 1 — Supplementary materials and methods [file PATH-252-88-s001.docx]

**Molecular and functional characterization of urine-derived podocytes from patients with Alport syndrome**

C Iampietro *et al. J Pathol* DOI: 10.1002/path.5496

**Supplementary materials and methods**

Reference numbers refer to the main text list

**Podocyte cell line generation and cloning**

Cells were infected with retroviral construct consisting of a Simian virus 40 large T (SV40T) and human telomerase reverse transcriptase vectors collected from the supernatants of the packaging cell line PA317 [41], carrying respectively geneticin (G418) and hygromycin resistance. Mid-confluent proliferating cells were exposed to freshly thawed, filtered (0.45 μm) supernatants mixed with growth medium plus 10 mg/ml polybrene at 37 °C. After 18–24 h, cells were cultured in growth medium at 33 °C in a 5% CO_2_ incubator and medium was changed every 2–3 days. Confluent cells were exposed to filtered (0.2 μm) culture medium supplemented with 400 μg/ml geneticin (Cat. No. 10131-035; Gibco–Thermo Fisher Scientific, Waltham, MA, USA) and 25 μg/ml hygromycin B (Cat. No. H0654; Sigma-Aldrich, St Louis, MO, USA) for 10–14 days at 33 °C until complete selection. Surviving cells were allowed to approach confluence in standard medium. For subcloning, cells were seeded at densities of 100 and 500 cells per 58 cm^2^ dishes at 33 °C in growth medium. Single cell clones were picked at 14–21 days by using trypsinized filters (Cat. No. Z374458-100EA-5mm; Sigma-Aldrich) and transferred to the individual wells of a 24-well plate. When they reached confluence, they were transferred to a larger flask and eight to nine clones for each patient were generated. Cells were grown at 33 °C to reach confluence; then were trypsinized and reseeded in fresh 75-cm^2^ flasks (300 000 cells per flask). After overnight incubation, cells were transferred to 37 °C to obtain fully differentiated podocytes (10–14 days) and medium was refreshed three times per week.

The following primers were used in qPCR:

| **Target** | **Forward primer** | **Reverse primer** |
| --- | --- | --- |
| *GAPDH* | CCGCTTCGCTCTCTGCTC | CGACCAAATCCGTTGACTCC |
| *SYNPO* | AGCCCAAGGTGACCCCGAAT | CCCTGTCACGAGGTGCTGGC |
| *PDX* | CTTGAGACACAGACACAGAG | CCGTATGCCGCACTTATC |
| *LMX1B* | AATGCAACCTGACCGAGAAG | ACATCATGCAGGTGAAGCAG |
| *VEGFA* | ATGAACTTTCTGCTGTCTTGGGTGC | TGATTCTGCCCTCCTCCTTCTGC |
| *COL4A1* | GTTGGTCTACCGGGACTCAA | GGCCTATTCCTGGAACTCCT |
| *COL4A3* | TACCAAGCCCACCACATGAT | AGTTGTAGCCAGCCGTACTT |
| *COL4A4* | GAACAAAAGGTGACCCAGGA | ATCCCCTTTTTCTCCAGC |
| *COL4A5* | TACTGGCCCTGAGTCTTTGG | CCTGGAGAACACCCATAGCA |
| *COL6A1* | CAGACACTCAGAGGGACACCAC | AAACACGTCTTTGATGCCCAC |
| *COL6A2* | CGAAGCCTACGGAGAGTGCTAC | CCTGTCTTCCCTTCTGGCC |
| *COL8A1* | CAGAAACCAGCCCCAGAGG | AATGGTAAGCAGCACTCCCAG |
| *MMP2* | CTGATAACCTGGATGCCGTCGT | TGCTTCCAAACTTCACGCTCTT |
| *LAMA5* | CCCACCGAGGACCTTTACTG | GGTGTGCCTTGTTGCTGTTG |
| *TALIN2* | CGGACACCTTATCGATCCCA | CACCAACTGCGGCTAAGAT |
| *SEMA5A* | ACTGTTCTAGCGACGGCACC | CCCCAGAAAGCCCATCTGT |
| *SEMA6A* | CCTGGACACCAGTTCCTGAT | CCCCAGAAAGCCCATCTGT |
| *RHOD* | CATGGTCAACCTGCAAGTGAA | CGCAGGCGGTCATAGTCAT |
| *WNT2B* | ATCTCATCAGCAGGGGTAGTCC | CAAAACGGACACCGTAGTGGA |
| *WNT10A* | GAGGCTTCACAACAACCGAGTT | CCGCATGTTCTCCATCACTG |
| *IGFBP5* | GTCCAAGTTTGTCGGGGGAG | GGGAAGGTTTGCACTGCTTT |
| *THBS1* | CATGCCACGGCCAACAA | GGCCCAGGTAGTTGCACTTG |
| *SERPINE1* | TTCAAGATTGATGACAAGGGCAT | CAGCCTGAAGAAGTGGGGC |

**Protein extraction and western blotting**

Proteins were quantified using Bradford solution according to the manufacturer’s procedures (Bio-Rad Inc, Berkeley, CA, USA). For western blotting, aliquots of cell lysates containing 30 µg of proteins were electrophoresed through 4–12% Mini-Protean TGX Stain-Free Gels (Bio-Rad) under reducing conditions. Using the iBLOT2 system (Life Technologies, Carlsbad, CA, USA), gels were blotted onto PVDF membrane filters according to the manufacturer’s procedures. Each membrane was immersed in blocking solution (5% milk powder in PBS) for 1 h before overnight incubation with primary antibodies at the indicated dilutions. After rinsing in wash buffer (0.1% Tween in PBS), horseradish peroxidase-conjugated secondary antibodies were used for 1 h at 1:3000–1:5000 dilutions. After final washings, membranes were incubated with ECL chemiluminescence reagent (Bio-Rad) and images acquired using a Chemidoc machine (Bio-Rad). For western blotting, the following antibodies were used: α-mouse vinculin (Cat. No. V4505; 1:8000 dilution; Sigma-Aldrich, St Louis, MO, USA), α-guinea pig nephrin (Cat. No. GP-N2; 1:500 dilution; Progen, Heidelberg, Germany), α-rabbit podocin (Cat. No. sc-21009; 1:200 dilution; Santa-Cruz, Dallas, TX, USA), α-goat synaptopodin (Cat. No. sc-21536; 1:200 dilution; Santa-Cruz), α-mouse WT-1 (Cat. No. sc-393498; 1:200 dilution; Santa-Cruz); and α-mouse GAPDH (Cat. No. ab37168; 1:1500; Abcam, Cambridge, UK).

**ELISA assays**

COL4α3 and COL3α5 protein expression levels were quantified in both cell lysates and cell supernatants using enzyme-linked immuno-sorbent assays (ELISA) (MyBioSource, San Diego, USA). Cell proteins were extracted from differentiated cells (13th or 14th day of differentiation) using Lysis Buffer (MyBioSource) following the manufacturer’s protocol. In brief, adherent cells were washed with cold PBS, trypsinized, and collected by centrifugation at 1000 × *g* for 5 min at room temperature. Then cells were washed three times in cold PBS, resuspended in fresh lysis buffer, and centrifuged at 1500 × *g* for 10 min at 4 °C to remove cell debris. Cell supernatants were collected after 72 h in cell culture, centrifuged at 1500 × *g* for 20 min to remove cell debris, and concentrated 40× by centrifugation at 2400 × *g* for 20 min at 4 °C using Amicon Ultra Centrifugal Filters (Cat. No. UFC903024; Merck Millipore, Darmstadt, Germany). Protein samples were quantified using Bradford’s methods (Bio-Rad) and assayed immediately or the day after collection. Data were expressed as concentration (ng/ml) of collagen 4 protein in total protein, constructing a four parameter logistic curve using MyAssays program (https://[www.myassays.com](http://www.myassays.com)). ELISA for VEGFA was performed using a VEGFA kit (Abcam) following the manufacturer’s instructions. For VEGFA protein evaluation in cell lysates, the same amount of total protein (10 µg) was used for each condition, while for cell supernatants, the VEGFA protein level was normalized to the total cell number for each condition.

**3D collagen IV models**

Models of collagen IV chains were obtained using *Phyre2* (<http://www.sbg.bio.ic.ac.uk/~phyre2/html/page.cgi?id=index>) and the altered amino-acid sequence was calculated with ExPasy (<https://web.expasy.org/translate/>) prediction algorithms. Due to the absence of a homologous structure for the low complexity regions and the repetitive collagen helix portion of the collagen type IV protein family, the only reliably predictable portion of the protein is the C-terminal type IV collagen C4 domains, which characterize all of the collagen type IV protein family. In order to avoid presentation of misleading models, we created the 3D models of the proteins limited to this highly reliable terminal portion.

**RNA sequencing and analysis**

RNA was isolated using the RNeasy mini kit (Qiagen, Hilden, Germany) and genomic DNA contaminations were removed with an Ambion^®^ DNA-free kit (Thermo Fisher Scientific). RNA concentration and quality were estimated with a NanoVuePlus Spectrophotometer (GE Healthcare, Chicago, IL, USA) and an Agilent 2100 Bioanalyzer (Agilent Technologies, Santa Clara, CA, USA). Libraries for RNA-seq were generated using a TruSeq RNA stranded sample preparation kit v2 (Illumina Inc, San Diego, CA, USA) following the manufacturer’s instructions, using 1 μg of total RNA as input material. Libraries were pooled and sequenced with a NextSeq 500 sequencer (Illumina Inc) generating 75-bp paired-end sequences. Demultiplexing was carried out using the bcl2fastq tool; expression quantification was carried out with STAR/RSEM using ENSEMBL annotation. Differential expression analysis was performed with the ANOVA-like comparing AS podocytes versus control urine-derived podocytes. Genes with a log_2_ FC ≥ 0.8; ≤ −0.8 and an adjusted *P* value ≤ 0.1 were considered as differentially expressed. Up-regulated genes underwent Gene Ontology (GO) enrichment analysis using TOPPGENE (<https://toppgene.cchmc.org/enrichment.jsp>). A hierarchical clustering heatmap was produced with G plots heatmap.2. Data were uploaded to the Gene Expression Omnibus database under accession number GSE134011.

**GEC purification**

Primary microvascular endothelial cells were obtained previously from cell outgrowths of human glomeruli, as described previously [22]. In brief, using sequential meshes, glomeruli were isolated from specimens of healthy human renal tissues removed for polar-confined carcinoma (after the approval of the ethical committee for the use of human tissue of Molinette Hospital; n. 168/2004). Glomeruli were digested by trypsin (0.1%, 30 min at 37 °C) and seeded onto gelatin in EndoGRO medium (Merck Millipore). After 7 days, cells were detached and purified by magnetic cell sorting using an anti-CD31 antibody in the MACS system (Miltenyi Biotec, Bergisch Gladbach, Germany). Subsequently, human glomerular endothelial cells (GECs) were characterized by morphology and expression of a panel of endothelial antigens, as described previously [22] (supplementary material, Figure S1). In particular, cells were analysed by cytofluorometric analysis using FACS Celesta (BD Biosciences, San José, CA, USA). Cells were detached using a non-enzymatic cell dissociation solution (Sigma) and resuspended in PBS 0.1% BSA (Sigma). The following antibodies conjugated to fluorescein isothiocyanate (FITC) or phycoerythrin (PE) were used for incubation: CD44 (BD); CD105, CD31, TIE-2, CD144, CD146 (Miltenyi Biotec).

**Immunofluorescence**

Immunofluorescence on cells co-cultured on inserts was performed as follows: after 48 h of co-culture, cells were fixed in 4% paraformaldehyde for 20 min at room temperature and permeabilized with 0.1% Triton X-100 (Sigma-Aldrich)/PBS for 10 min at 4 °C. Non-specific sites were blocked with 1.5% bovine serum albumin (BSA; Sigma-Aldrich)/PBS for 20 min at room temperature. FITC-αSMA (Sigma-Aldrich) was incubated for 1 h at 1:500 dilution. Fixed cells were washed with 0.1% BSA/PBS before nuclear staining with Hoechst 33258 dye (Sigma-Aldrich) at 1:10 000 dilution for 8 min. After the final wash, coverslips were mounted with Fluoromount. Imaging was performed using a Leica TCS SP5 confocal system (LEICA Microsystems S.r.l., Wetzlar, Germany) equipped with a 405 nm diode and argon ion lasers. Samples were imaged using a 40X PlanApo/1.4 NA oil immersion objective. A series of *x-y-z* images (typically 0.19 * 0.19 * 0.5 µm voxel size) were collected along the *z*-axis at 0.5 µm intervals throughout the sample depth (36 µm). 3D volumes and movies were generated using LasX software (LEICA Microsystems S.r.l.). 3D reconstruction was performed using ‘volume rendering’. The surface of the co-culture was created using the ‘surface’ tool by creating a mask around each volume. 3D images and movies were obtained adopting the ‘snapshot’ and ‘movie creator’ tools.

Immunofluorescence was also performed on single podocyte cultures for the evaluation of collagen IV α5 chains. Podocytes were plated onto slides (Thermo Fisher Scientific) and kept in differentiation conditions at 37 °C for 14 days, fixed, and permeabilized as above. Podocytes were incubated with rabbit polyclonal anti-COL4A5 (Cat. No. sc-11360; dilution 1:1000; Santa-Cruz) for 1 h at room temperature, followed by AlexaFluor 488-labeled goat anti-rabbit secondary Ab (Cat. No. A11008; dilution 1:1000; Invitrogen, Carlsbad, CA, USA) at room temperature for 1 h. Texas Red-X Phalloidin (Cat. No. T7471; dilution 1:1000; Thermo Fisher Scientific) was added to the secondary antibody solution and cells were examined using a Leica TCS SP5 confocal system (LEICA Microsystems S.r.l.) equipped with an argon ion and 561 nm lasers. Samples were imaged using a 40X PlanApo/1.4 NA oil immersion objective. A series of *x-y-z* images (typically 0.75 * 0.75 * 1 µm voxel size) were collected.

**Millifluidic system**

For all co-culture experiments, first GECs were seeded on the lower PET membrane side at a density of 8 × 10^4^ cells per 12-well insert (ipCELLCULTURE^TM^ Track Etched Membrane; 0.45 µm pore size; it4ip S.A., Louvain-la-Neuve, Belgium) and after 6 h, podocytes were seeded on the upper side at the same density. Each cell type was cultured in its growth medium for 48 h before permeability assay and medium was changed once the day after cell plating. To confirm that no difference in cell number was present, we validated our system by counting cell detachment of differentiated cells after 48 h. The percentage of cell detachment after 48 h was 7.1% and 10.0% in control and AS, respectively. This number was taken into consideration when plating cells for experiments in order to gain a complete monolayer (8 × 10^4^ cells per 12-well insert, as detailed in the Results section). For dynamic experiments, we used a dynamic system fabricated by IVTech Srl (Lucca, Italy). This millifluidic device allows continuous perfusion for live cells seeded in a chamber called LiveBox (LB). The main body of the device, the LiveFlow (LF), is composed of two pumping heads, each one driving two independent circuits. A circuit is in turn made up of three devices connected to each other: a peristaltic pump able to apply a specific flow rate to the fluid in the system; LB, which allows an *in vitro* physiological barrier to be recreated; and tanks containing liquids entering and leaving the system. LB is a silicone chamber with two compartments separated by a porous membrane: one lower compartment for GECs and one upper compartment for podocytes. In short, podocyte medium contained in one of the two inlet tanks flows up to the peristaltic pump, which gives a flow rate of 100 µl/min to the fluid that arrives in the LB upper part, where the podocytes are present. In the same way, an endothelial culture medium is conveyed into the inferior part of the room. The fluid leaving each compartment of the culture box is collected separately in two tanks (Figure 6A). During the permeability test, cells in the chamber are subjected to a shear stress of 8 × 10^−5^ dyn/cm^2^. To roughly calculate this value, we used the following equation: τ = 6µ*Q*/*bh*^2^, where µ is the medium viscosity (g/cm per s), *Q* is the volumetric flow rate (cm^3^/s), *b* is the channel width, and *h* is the channel height [42–44].
