# Supplementary material for: Molecular and functional characterization of urine‐derived podocytes from patients with Alport syndrome
Source: J Pathol. 2020 Aug 19;252(1):e5496. doi: 10.1002/path.5496 (PMC7589231; doi:10.1002/path.5496)
Supplement: Supplementary file 2 — Figure S1. Glomerular endothelial cell (GEC) characterization Figure S2. Immunofluorescence microscopy for COL4α5 protein Figure S3. RT‐PCR for COL4A3 and COL4A5 transcripts Figure S4. Connectome of differentially expressed genes in AS podocytes [file PATH-252-88-s002.docx]

**Molecular and functional characterization of urine-derived podocytes from patients with Alport syndrome**

C Iampietro *et al. J Pathol* DOI: 10.1002/path.5496

**Supplementary Figures S1–S4**

**Supplementary Video S1 legend**


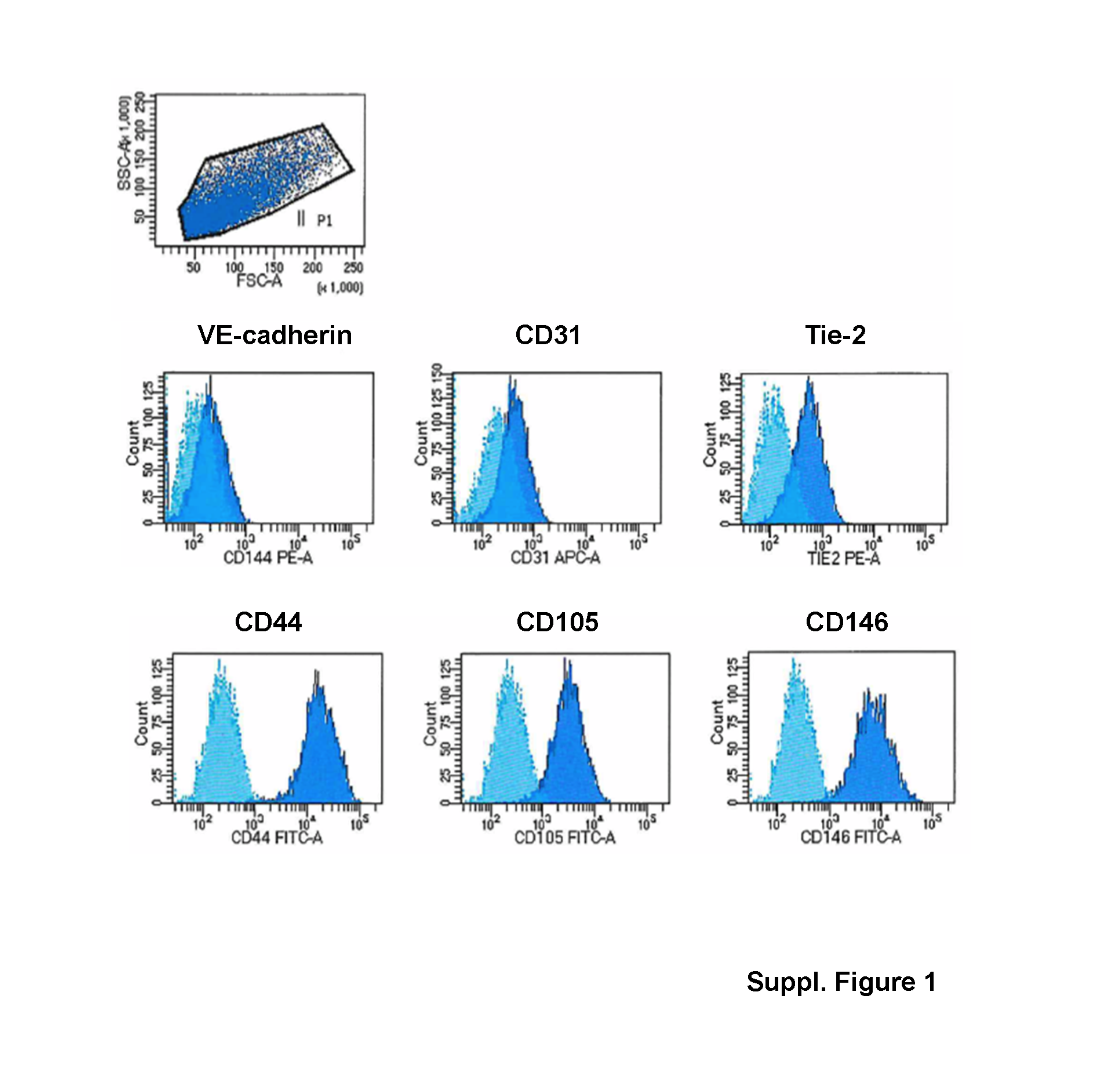


**Figure S1. Glomerular endothelial cell (GEC) characterization.**

Representative cytofluorimetric analysis showing the expression of endothelial markers by GECs. The blue area shows binding of the specific antibody and the turquoise area the isotypic control. All lines in study (*n* = 3) showed similar marker expression.


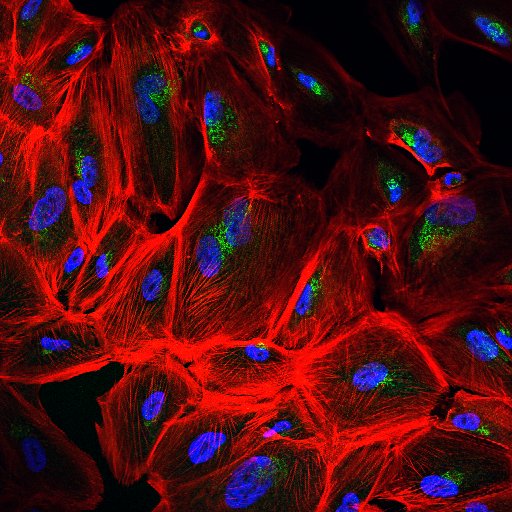

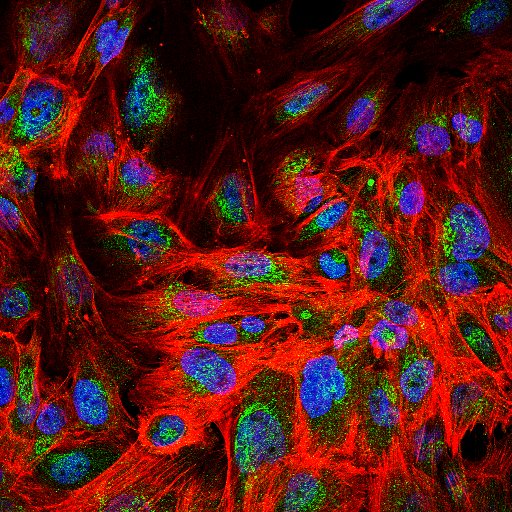


K-pod

AS patient 3

**Col4α5**

**PH**

**DAPI**

**Col4α5**

**PH**

**DAPI**

10 µm

10 µm

**Figure S2. Immunofluorescence microscopy for COL4α5 protein.**

Differentiated K-pod (left) and AS patient 3 podocytes (right) were stained for COL4α5 (green), phalloidin (red), and nuclear staining was performed with Hoechst dye 33342. Original magnification ×400.

**
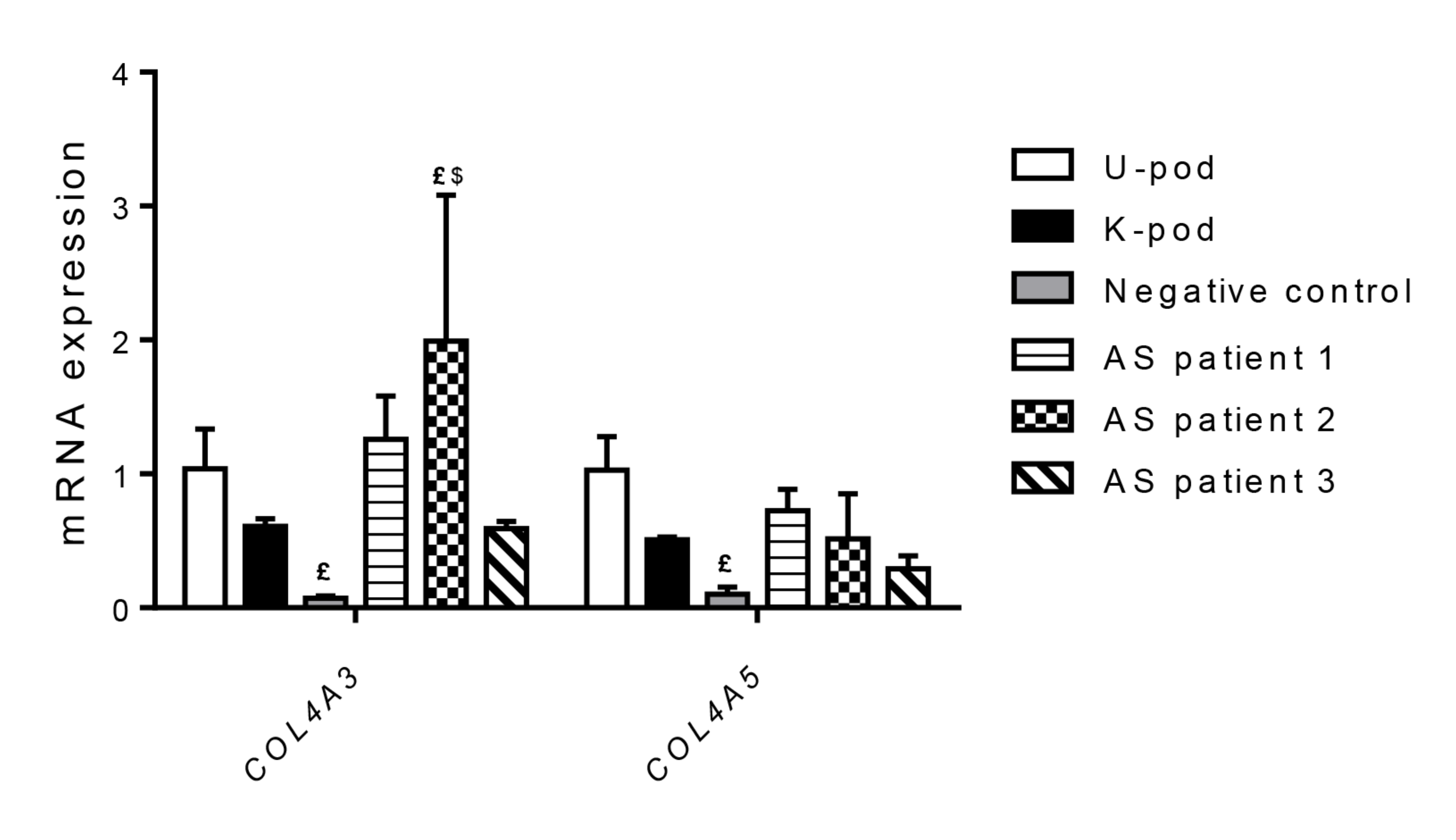
**

**Figure S3. RT-qPCR for *COL4A3* and *COL4A5* transcripts.**

Data are shown as relative quantification, normalized to *GAPDH* and to control kidney-derived podocytes (K-pod). Three clones for each AS patient were analyzed and data are expressed as mean ± SD. GECs were used as a negative control. ^£^*p* < 0.05 versus U-pod; ^$^*p*< 0.05 versus K-pod.

**
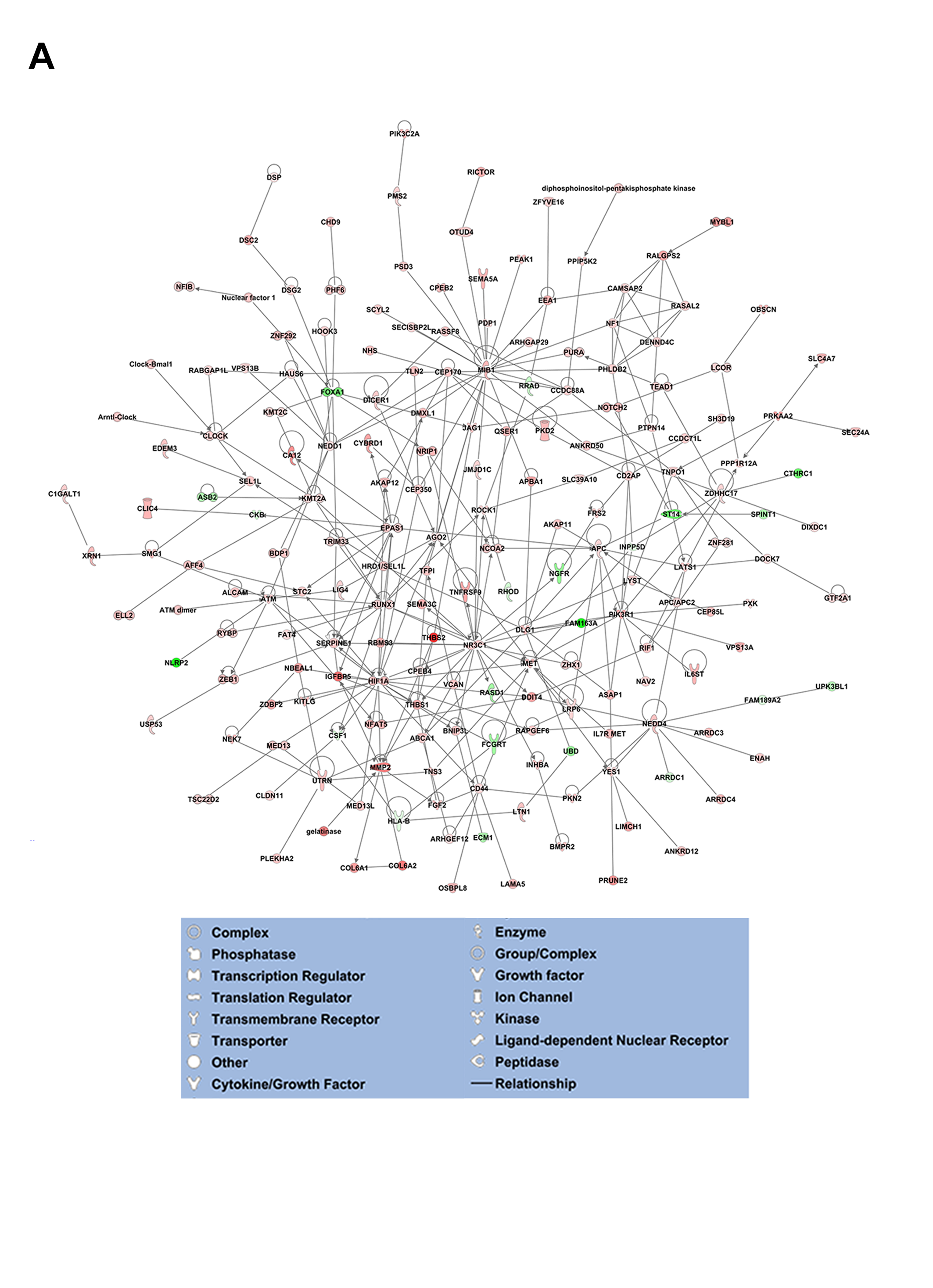
**

**Figure S4. Connectome of differentially expressed genes in AS podocytes.** Ingenuity Pathway Analysis (IPA, Qiagen) network of the differentially expressed genes of AS podocytes (three clones per patient) compared with control urine-derived podocytes.

**Video S1. 3D reconstruction of a podocyte–GEC co-culture.**

The video shows a 3D reconstruction of immunofluorescence-visualized co-cultures performed using ‘volume rendering’. Cells were stained with phalloidin (green) and nuclear staining (blue) was performed with Hoechst dye 33342. The surface of the co-culture was created using the ‘surface’ tool by creating a mask around each volume. 3D image and the movie were obtained adopting the ‘snapshot’ and ‘movie creator’ tools and were generated using LasX software (LEICA Microsystems S.r.l.). Original magnification: ×400.
